# Antinociceptive effect of cyclic phosphatidic acid and its derivative on animal models of acute and chronic pain

**DOI:** 10.1186/1744-8069-7-33

**Published:** 2011-05-14

**Authors:** Yasutaka Kakiuchi, Jun Nagai, Mari Gotoh, Harumi Hotta, Hiromu Murofushi, Tomoyo Ogawa, Hiroshi Ueda, Kimiko Murakami-Murofushi

**Affiliations:** 1 Department of Biology, Faculty of Science, Ochanomizu University, 2-1-1 Ohtsuka, Bunkyo-ku, Tokyo 112-8610, Japan; 2 Division of Molecular Pharmacology and Neuroscience, Nagasaki University Graduate School of Biomedical Sciences, 1-14 Bunkyo-machi, Nagasaki 852-8521, Japan; 3 Department of Autonomic Neuroscience, Tokyo Metropolitan Institute of Gerontology, 35-2 Sakae-cho, Itabashi-ku, Tokyo 173-0015, Japan

## Abstract

**1. Abstract:**

**Background**

Cyclic phosphatidic acid (cPA) is a structural analog of lysophosphatidic acid (LPA), but possesses different biological functions, such as the inhibition of autotaxin (ATX), an LPA-synthesizing enzyme. As LPA is a signaling molecule involved in nociception in the peripheral and central systems, cPA is expected to possess analgesic activity. We characterized the effects of cPA and 2-carba-cPA (2ccPA), a chemically stable cPA analog, on acute and chronic pain.

**Results**

(1) The systemic injection of 2ccPA significantly inhibited somato-cardiac and somato-somatic C-reflexes but not the corresponding A-reflexes in anesthetized rats. (2) 2ccPA reduced sensitivity measured as the paw withdrawal response to electrical stimulation applied to the hind paws of mice through the C-fiber, but not Aδ or Aβ. (3) In mice, pretreatment with 2ccPA dose-dependently inhibited the second phase of formalin-induced licking and biting responses. (4) In mice, pretreatment and repeated post-treatments with 2ccPA significantly attenuated thermal hyperalgesia and mechanical allodynia following partial ligation of the sciatic nerve. (5) In rats, repeated post-treatments with 2ccPA also significantly attenuated thermal hyperalgesia and mechanical allodynia following chronic sciatic nerve constriction.

**Conclusions**

Our results suggest that cPA and its stable analog 2ccPA inhibit chronic and acute inflammation-induced C-fiber stimulation, and that the central effects of 2ccPA following repeated treatments attenuate neuropathic pain.

## 2. Background

Cyclic phosphatidic acid (cPA) was originally isolated from myxoamoebae of a true slime mold, *Physarum polycephalum*, in 1992 [1]. The chemical formula of cPA is similar to that of lysophosphatidic acid (LPA), but cPA has a unique structure with a cyclic phosphate ring at *sn*-2 and *sn*-3 of the glycerol backbone [2]. These features provide cPA with distinct/opposing biological functions from those of LPA. For instance, LPA stimulates cell proliferation and cancer cell invasion, while cPA inhibits these activities [3-8]. Interestingly, LPA is enzymatically generated from transphosphatidylation of lysophosphatidylcholine by autotaxin (ATX) [9], but cPA inhibits ATX activity [10]. Thus, cPA could be an endogenous inhibitor of LPA production through ATX.

Exogenous and endogenous LPA cause acute pain through C-fibers and neuropathic pain [11-13]. Recent studies revealed that nerve injury-induced LPA production and neuropathic pain were significantly attenuated in mice with heterozygous ATX deficiency [14,15]. In this study, we characterized the effects of cPA and its chemically stable analog 2ccPA on acute and neuropathic pain.

## 3. Methods

### 3.1. Recording the somato-cardiac sympathetic reflex

The experiments were performed using male Wistar rats (n = 11) weighing 270-370 g anesthetized by intraperitoneal (i.p.) application of urethane (1.1 g/kg). A femoral vein was catheterized for intravenous (i.v.) administration of supplemental anesthetics and cPA. A femoral artery was catheterized to record arterial blood pressure and heart rate. The animals were immobilized by gallamine triethiodide (20 mg/kg i.v. as required) and artificially ventilated via tracheal cannula. Ventilation was monitored with a gas analyzer (1H26; NEC San-ei, Tokyo) and adjusted to maintain an end-tidal CO_2 _level of 3.0%. Body temperature was maintained at 37.5°C using an automatically regulated heating pad and lamp (ATB-1100; Nihon Kohden, Tokyo). Both vagal nerves were cut at the cervical level to prevent vagal contamination of the recorded sympathetic nerve activity.

With the rat placed in the supine position, the right second costal bone was removed. The right inferior cardiac sympathetic nerve was dissected retropleurally, cut as close to the heart as possible, and covered with warm paraffin oil. Cardiac sympathetic efferent nerve activity was recorded from the central segment of the cardiac sympathetic nerve with platinum-iridium wire electrodes using an AC preamplifier (S-0476; Nihon Kohden, Tokyo; time constant set at 0.33 s). Reflex responses elicited by electrical stimulation of a hind limb nerve were averaged (50 trials) by a computer (ATAC 3700; Nihon Kohden, Tokyo or UPO, Unique Medical, Tokyo). The averaged responses were stored as digital signals and recorded on a printer or mini-writer. The size of the reflex response was measured as the area under the evoked response and expressed as the percent of the control size preceding drug injection.

A tibial nerve was dissected from the surrounding tissues and cut. The central cut end segment of the nerve was placed on bipolar platinum-iridium wire electrodes for electrical stimulation. Single square pulse stimuli of 0.5 ms duration were delivered every 3 s by a digital electrical stimulator (SEN-7103; Nihon Kohden, Tokyo).

### 3.2. Recording spinal somato-somatic reflex

Male Wistar rats (n = 4; weighing 330-350 g) were anesthetized using pentobarbital (50 mg/kg i.p.). A jugular vein was catheterized for i.v. administration of cPA. Body temperature was maintained at 37.5°C. The spinal cord was completely transected at the upper thoracic level.

With the rat placed in the supine position, a branch of the right saphenous nerve innervating thigh skin was cut at the thigh level. The central cut end segment of the nerve was stimulated electrically as described above. Electromyogram (EMG) was recorded from the right leg muscles by inserting silver electrodes using the AC preamplifier. With single shocks, post-stimulus time histograms were created by a computer (ATAC 3700; Nihon Kohden, Tokyo) for EMG activity for approximately 50 trials at 3-s intervals. The averaged responses were stored as digital signals and recorded on a mini-writer. The size of the reflex response was measured as the area under the evoked response and expressed as the percent of the control size preceding drug injection.

### 3.3. Electrical stimulation-induced paw withdrawal (EPW) Test

Male C57BL/6 mice (TEXAM, Japan) weighing 18-22 g were used. The electrical stimulation-induced paw withdrawal (EPW) test conducted using the Neurometer™ CPT/C (Neurotron Inc.) has been reported previously [16]. In brief, electrodes were fastened to the plantar surface and instep of the mice. Transcutaneous nerve stimuli with each of the 3 sine wave pulses (5, 250, and 2000 Hz) were applied using the Neurometer™. The minimum intensity at which each mouse exhibited paw withdrawal was defined as the current stimulus threshold at 10-15 min after i.v. 2ccPA injection.

### 3.4. Formalin test

According to several preceding works [17,18] and to an economical advantage, we used ICR (CD1) female mice (Charles River, Japan), 6-9 weeks of age for the formalin test. Formalin solution (30 μL, 2% v/v) in saline was subcutaneously (s.c.) injected into the plantar surface of the left hind paw. Immediately after the formalin injection, the animals were placed in a cage and videotaped for 30 min from beneath the transparent floor. The time (in seconds) spent licking and biting the injected paw was counted in 5-min intervals by videotape observation. Two distinct phases of intensive licking and biting activities identified as the early and late phases were defined at 0-10 min and 10-30 min, respectively. At 3 min before formalin was injected, 2ccPA solution (in PBS containing 1% BSA) was i.v. injected. Morphine hydrochloride solution (Takeda Pharmaceutical Company, Osaka, Japan) in saline was i.p. injected at 30 min before formalin injection.

### 3.5. Neuropathic pain models

In experiments using the C57BL/6 mice, partial ligation of the sciatic nerve was performed under pentobarbital (50 mg/kg) anesthesia, following the methods of Malmberg and Basbaum [19]. 2ccPA was dissolved in artificial cerebrospinal fluid (aCSF: 125 mM NaCl, 3.8 mM KCl, 1.2 mM KH_2_PO_4_, 26 mM NaHCO_3_, 10 mM glucose, pH 7.4). The intrathecal injection (i.t.) of 2ccPA was given into the space between spinal L5 and L6 segments according to the method of Hylden and Wilcox [20]. In the thermal paw withdrawal tests, nociception was measured as the latency to paw withdrawal evoked by exposure to a thermal stimulus [21,22]. Unanesthetized animals were placed in Plexiglas cages on the top of a glass sheet and were allowed an adaptation period of 1 h. A thermal stimulator (IITC Inc., Woodland Hills, CA, USA) was positioned under the glass sheet, and the focus of the projection bulb was aimed precisely at the middle of the plantar surface of the animal. A mirror attached to the stimulator permitted plantar surface visualization. The paw pressure test was performed, as described previously [22]. Mice were placed into a Plexiglas chamber on a 6 × 6 mm wire-mesh grid floor and allowed to acclimatize for 1 h. A mechanical stimulus was delivered onto the middle of the plantar surface of the right hind paw using a Transducer Indicator (Model 1601; IITC Inc., Woodland Hills, CA, USA). The pressure needed to induce a flexor response was defined as the pain threshold.

In experiments using rats, the chronic constriction injury was produced according to the procedure of Bennett and Xie [23]. Briefly, rats (182-216 g at day of ligation) were anesthetized with sodium pentobarbital (Nembutal, Dainippon pharmaceutical co., 40 mg/kg i.p.). The left sciatic nerve was exposed at mid-thigh level. Three loose ligatures with 4.0 chromic gut (SG-535; Syneture, USA), about 1-mm spacing, were tied around the sciatic nerve proximal to the trifurcation. Six days after nerve ligation, the development of neuropathy was assessed by measuring paw withdrawal latencies against thermal and mechanical stimuli [24,25]. Briefly, the thermal withdrawal threshold of a hind paw was measured using a beam of radiant infrared heat and a photocell (Planter Test model 7370; Ugo Basil, Milan, Italy). To prevent tissue damage, the cut-off time was set to 20 or 30 sec for rats with or without ligation. The mechanical withdrawal threshold was measured by the von Frey filament test. All rats were administered drug treatment by i.v. injection, and experiments were performed at 10 min and at 2 and 4 hours after drug treatments.

### 3.6. Drugs

**Figure 1:**
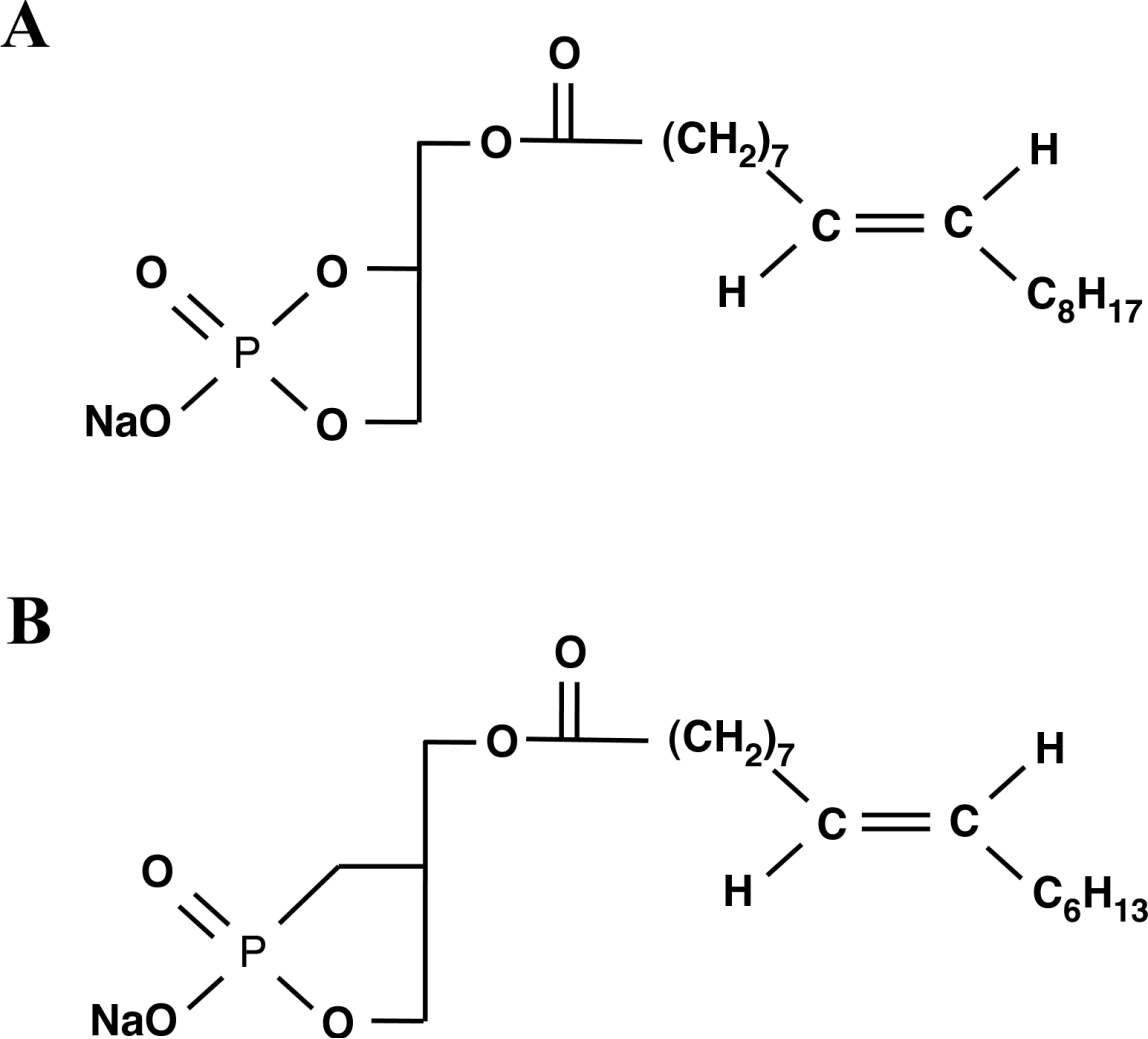
**Structure of cPA and 2ccPA**. A naturally occurring derivative (A; cPA 18:1) and a chemically synthesized derivative used for the present experiments (B; 2ccPA 16:1).

We used chemically synthesized cPA 18:1 and its biologically stable derivative, 2-carba cPA 16:1 (2ccPA) (Figures 1A, B) [6]. These compounds were i.v. or i.t administered after they were dissolved in saline or artificial cerebrospinal fluid (aCSF).

### 3.7. Statistical analysis

Data were expressed as mean ± S.E. The data were statistically analyzed by Student's t-test, Welch's test, Steel's test, Dunnett's test, Wilcoxon test, or Tukey's multiple comparison tests. A P value < 0.05 was considered statistically significant.

### 3.8. Ethical approval

All the experiments were performed with an approval of the Animal Care and Use Committee at the Tokyo Metropolitan Institute of Gerontology and Nagasaki University Animal Care Committee at the Nagasaki University Graduate School of Biomedical Sciences (reference number 0706130596).

## 4. Results

### 4.1. Effects of cPA and 2ccPA on somato-cardiac sympathetic A- and C-reflexes in anesthetized rats

**Figure 2:**
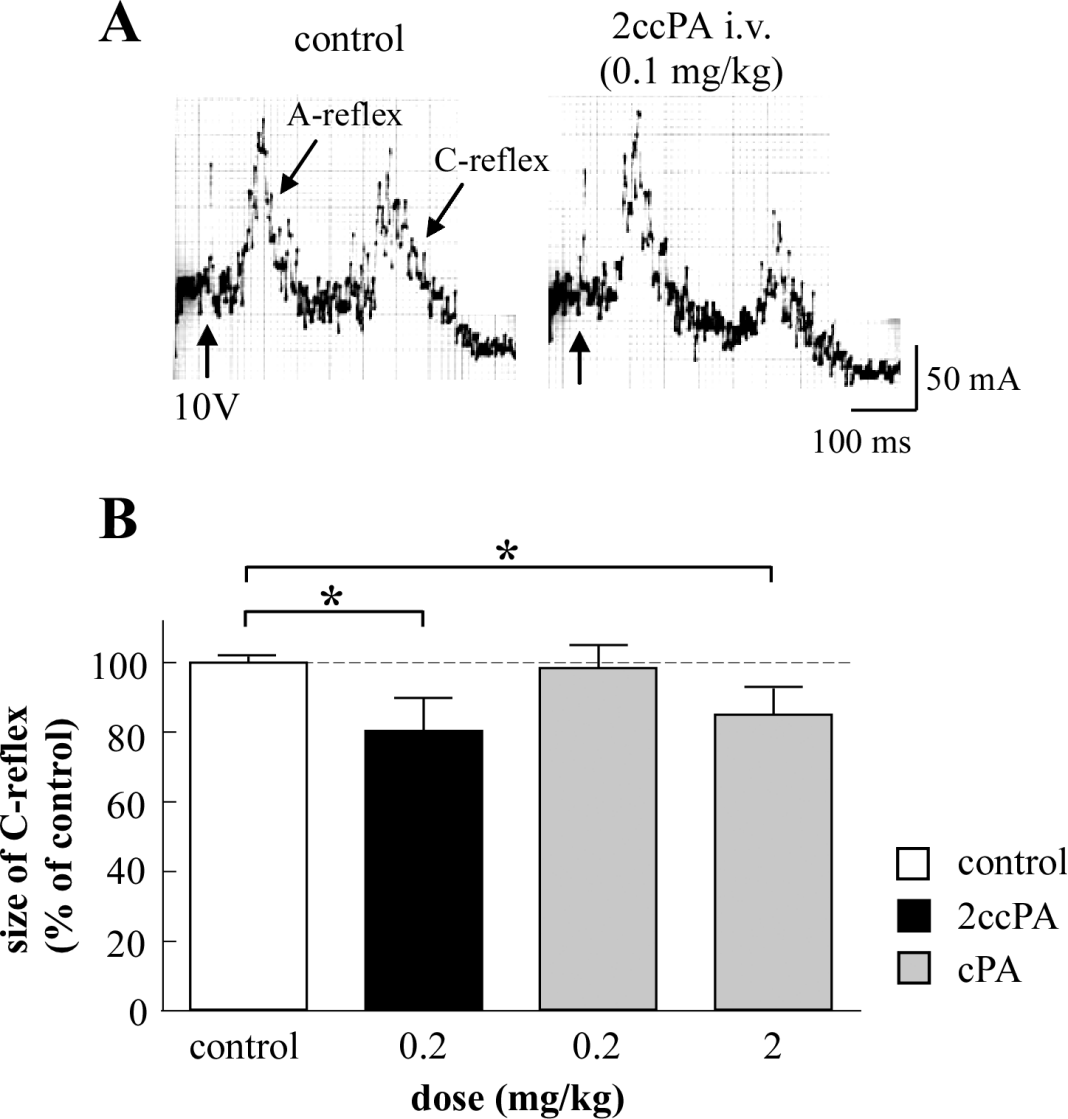
**Effects of i.v. cPA and 2ccPA on somato-cardiac sympathetic A- and C-reflexes**. A, Specimen records of A- and C-reflexes (averages of 50 trials) elicited by single electrical stimulation of a tibial nerve using 10 V of stimulus strength. Left: prior to 2ccPA application (control). Right: 15 min after i.v. injection of 0.1 mg/kg 2ccPA. B, Graphical summary of the size of the C-reflex at 0-5 min before cPA/2ccPA injection (control; n = 8), at 10-15 min after i.v. injection of 0.2 mg/kg 2ccPA (n = 4), and 0.2 mg/kg (n = 4) and 2 mg/kg (n = 4) cPA. We averaged 1-2 trials for each rat. The mean size (area under the evoked response curve) of the C-reflex at 0-10 min before each injection was expressed as 100%. All subsequent reflexes were expressed as percentages of the control values. Each column and vertical bar represent mean and S.E. *P < 0.05; significantly different from the control response by Student's t-test.

Single shock stimulation of A- and C-afferent fibers of the tibial nerve (at 10 V with 0.5 ms pulse duration) elicited 2 types of cardiac sympathetic reflex discharges as shown in Figure 2A. These discharges occur in a short latency (about 40 ms) as the A-sympathetic reflex and in a long latency (about 210 ms) as the C-sympathetic reflex, respectively (Figure 2A).

Intravenous injection of 0.1 mg/kg of 2ccPA depressed the C-reflex but not the A-reflex (Figure 2A). In most experiments, the 2ccPA-induced depression of C-reflex components started several minutes after the injection, reached its maximum level in less than 15 min, and then gradually returned to baseline within 40 min. The response was reproducible in successive trials on the same animal. Injection of 2ccPA 18:1 also yielded similar C-reflex suppression (data not shown).

Figure 2B summarizes the effects of i.v. injection of 2ccPA and cPA on the C-reflex in 8 rats. The responses were measured at 10-15 min after i.v. injection of cPA, i.e., after reaching the maximum effect (see above). After injection of 0.2 mg/kg 2ccPA, the C-reflex reached 82 ± 9% of the control. Injection of 2 mg/kg, but not 0.2 mg/kg cPA, significantly depressed the C-reflex. After injection of 2 mg/kg cPA, the C-reflex reached 86 ± 8% of the control.

### 4.2. Effects of cPA and 2ccPA on somato-somatic A- and C-reflexes in anesthetized rats

**Figure 3:**
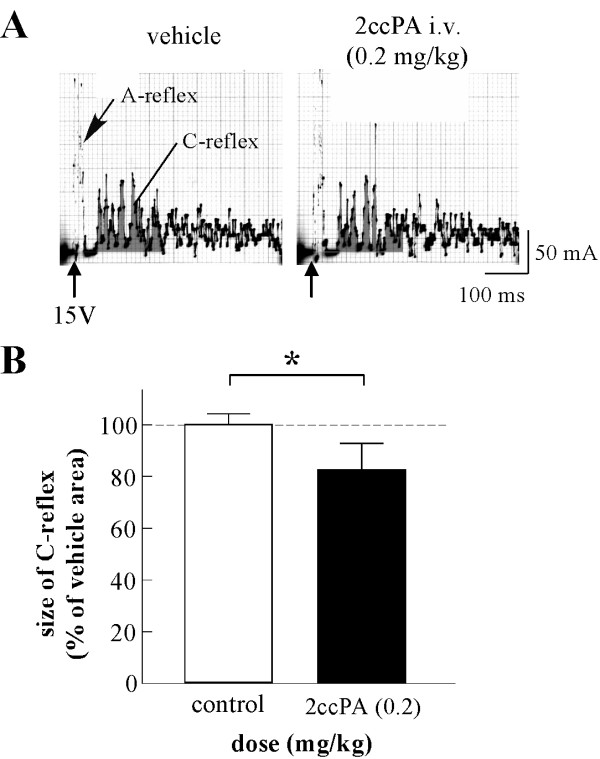
**Effects of i.v. 2ccPA on the somato-somatic C-reflex**. A, Specimen records of A- and C-reflexes (averages of 50 trials) elicited by single electrical stimulation of a saphenous nerve using 15 V of stimulus strength. Left: prior to 2ccPA application (control). Right: 10 min after i.v. injection of 0.2 mg/kg 2ccPA. B, Graphical summary of the size of the C-reflex at 0-5 min before 2ccPA injection (control; n = 4) and at 10-15 min after i.v. injections of 0.2 mg/kg 2ccPA (n = 4). We averaged 1-2 trials for each rat. The mean size (area under the evoked response curve) of the C-reflex at 0-10 min before each injection was expressed as 100%. All subsequent reflexes were expressed as percentages of the control values. Each column and vertical bar represent mean and S.E. *P < 0.05; significantly different from the control response by Student's t-test.

The hypothesis that cPA acts at the primary afferent nerve terminals was tested by recording spinal somato-somatic reflexes in 4 rats (Figure 3). Stimulation of the myelinated A-afferent fibers and unmyelinated C-afferent fibers of the saphenous nerve (at 15 V with 0.5 ms pulse duration) produced 2 distinct A- and C-somatic reflex components in the hind limb EMG, i.e., a short latent (about 10 ms) A-reflex and long latent (about 70 ms) C-reflex. Intravenous application of the same 2ccPA dose (0.2 mg/kg) that depressed the somato-sympathetic C-reflex also depressed the C-somatic reflex component to a similar magnitude. After injection of 0.2 mg/kg 2ccPA, the C-reflex reached 83 ± 5% (n = 4) of the control (p < 0.05).

### 4.3. C-fiber specific analgesic effects of 2ccPA on electrical stimulation-induced paw withdrawal (EPW)

**Figure 4:**
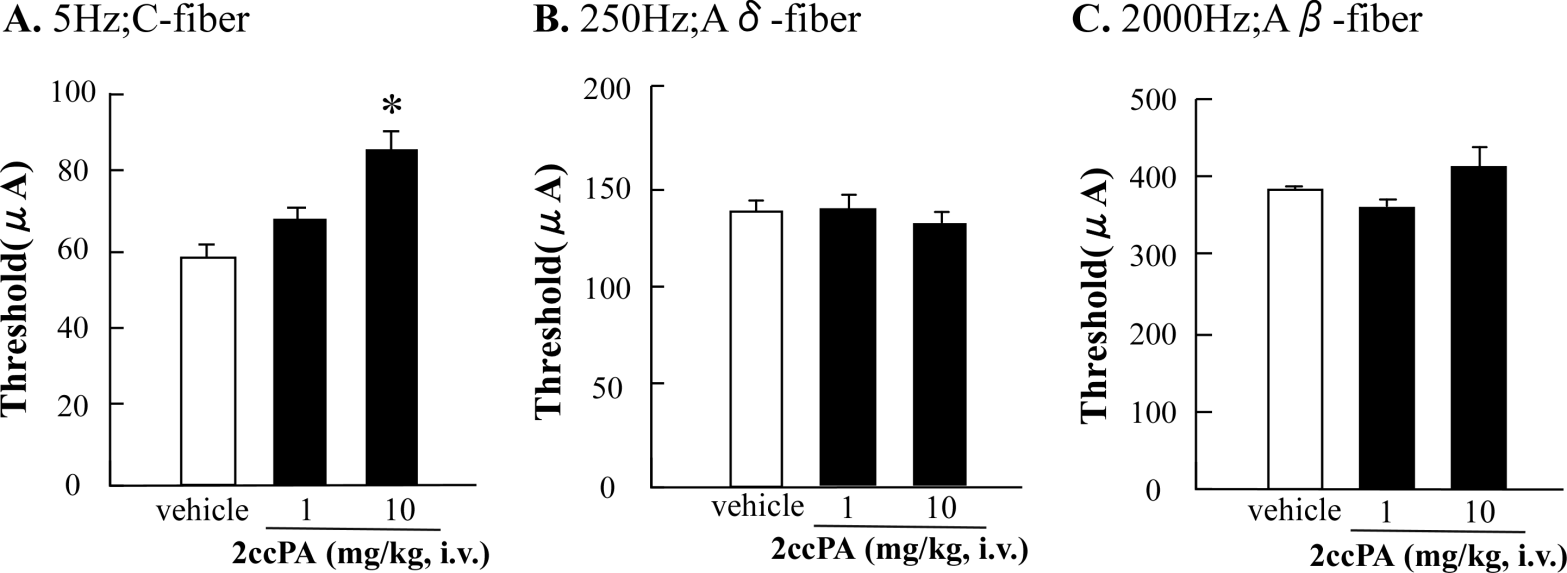
**Effects of cPA on the Electrical Stimulation-Induced Paw Withdrawal (EPW) test**. The threshold represents the minimum intensity (μA) required to elicit a paw withdrawal response to electrical stimulation with 5 Hz (C-fiber) (A), 250 Hz (Aδ-fiber) (B), and 2000 Hz (Aβ-fiber) (C). The EPW test was performed 10-15 min after 2ccPA (i.v.) injection. All data represent the mean ± S.E. from 3-6 individual mice per group. *P < 0.05; significantly different from the control response by Tukey's multiple comparison.

We established the EPW test to distinguish responses mediated by different sensory fibers [16]. The hind paw is given transcutaneous nerve stimuli with sine-wave pulses of 5, 250, or 2000 Hz to stimulate C-, Aδ-, or Aβ-fibers [26,27], and the intensity (μA) required to induce a withdrawal reflex was defined as the threshold. The thresholds of naïve mice for 5, 250, and 2000 Hz stimuli were 64.4 ± 1.9 (C-fibers), 145.0 ± 4.5 (Aδ-fibers), and 401.1 ± 13.6 (Aβ-fibers). When the EPW test was performed at 10-15 min after the 2ccPA (i.v.) injection, the threshold increased in a dose-dependent manner with 5 Hz, but it did not increase with 250- or 2000-Hz stimuli (Figure 4).

### 4.4. Analgesic effects of 2ccPA on mouse formalin-evoked licking and biting behavior

**Figure 5:**
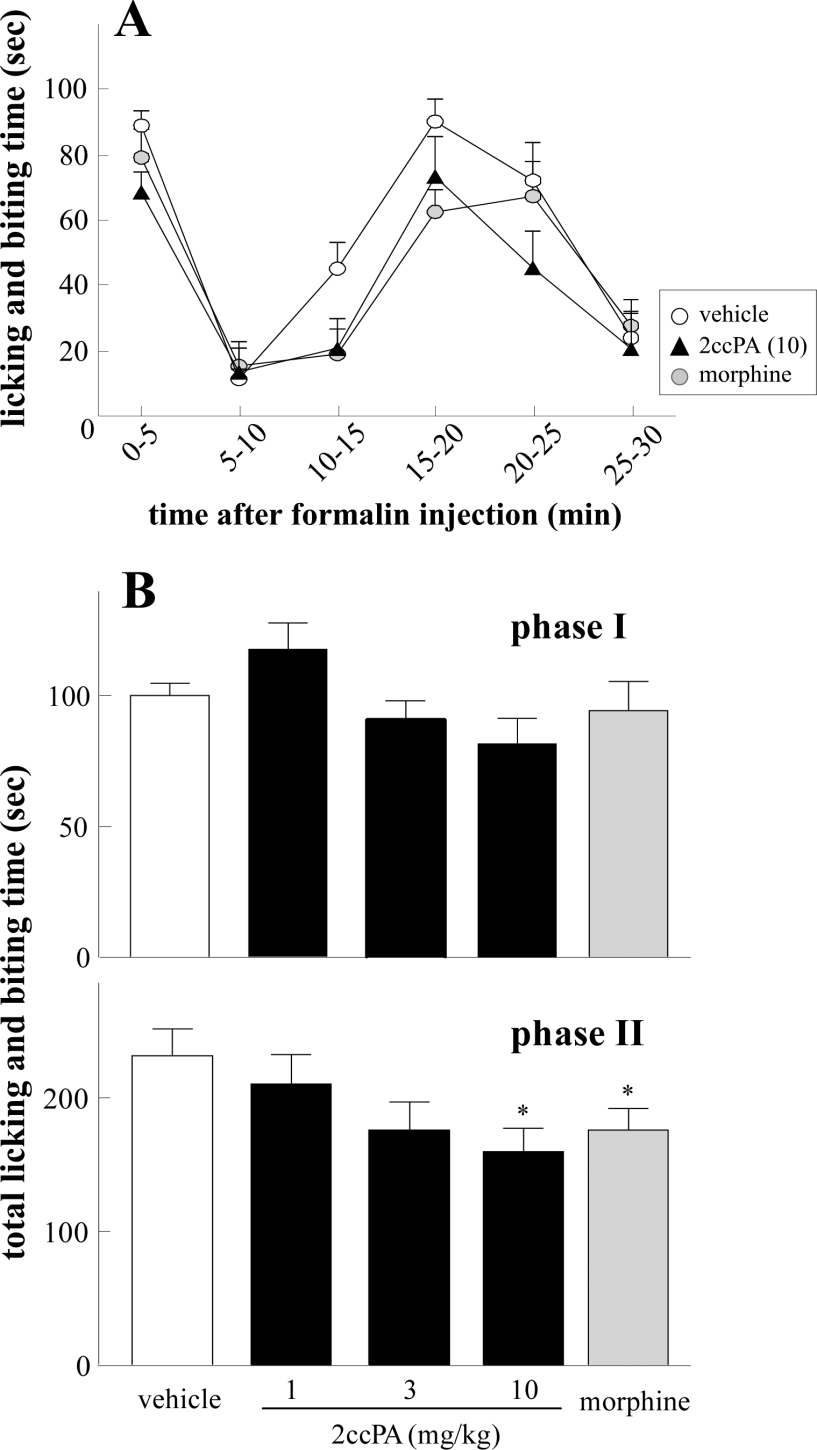
**Effects of i.v. 2ccPA on licking and biting time**. A, Time variations of licking and biting time of mice s.c. injected with 30 μL of 2% formalin in the mouse hind paw immediately after i.v. injection with 10 mg/kg (closed triangle) 2ccPA or vehicle (open circle). Morphine (3 mg/kg i.p.) was applied 30 min prior to formalin injection as a control. Each data point represents the average licking and biting time for 8-13 mice. B, Graphical summary of the total phase I and II licking and biting times of mice at 1 mg/kg, 3 mg/kg, or 10 mg/kg 2ccPA. All data represent the mean ± S.E. *P < 0.05; significantly different from the control response by Student's t-test.

In the formalin-induced nociception tests, ICR (CD1) mice were given an s.c. injection of formalin solution to the hind paw. As shown in Figure 5A, the time course of the total time spent in licking and biting comprised 2 phases. Morphine or i.v. injection of 2ccPA at 30 min prior to formalin injection markedly reduced phase II licking and biting. Quantitative analysis revealed that 2ccPA exerted dose-dependent inhibition of phase II responses, with significant inhibition observed at a 10-mg/kg i.v. dose (Figure 5B). As a reference, significant analgesia was also achieved with 3 mg/kg morphine.

### 4.5. Pre-injury administration of 2ccPA prevents neuropathic pain development

**Figure 6:**
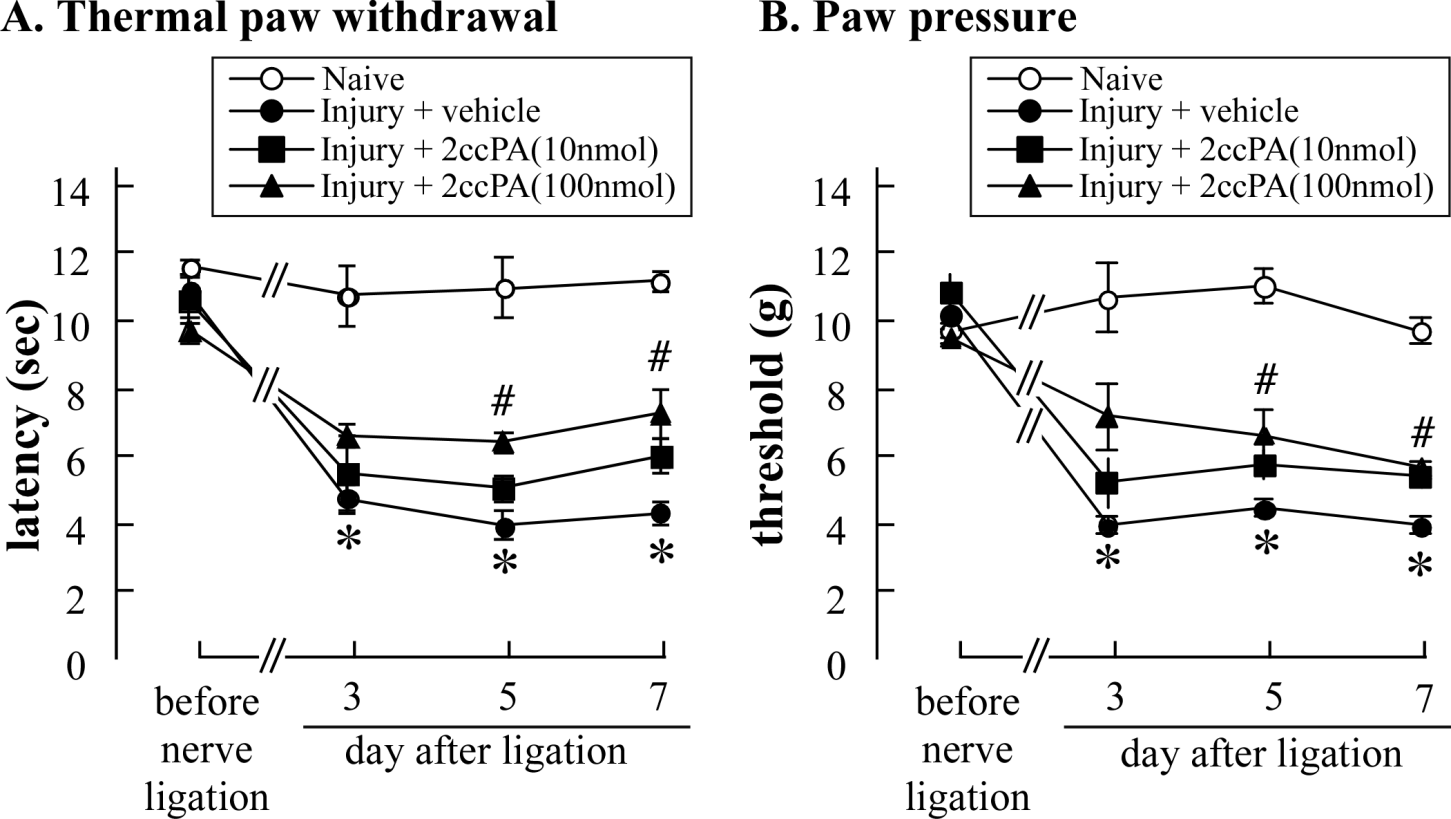
**Pre-injury administration of 2ccPA (i.t.) prevents development of neuropathic pain**. Neuropathic pain was induced by partial sciatic nerve injury in mice. 2ccPA (10 or 100 nmol/5 μL i.t.) was injected at 30 min prior to nerve injury. The threshold was measured on days 3, 5, and 7 after nerve injury, using the thermal withdrawal (A) and paw-pressure (B) tests. All data represent the mean ± S.E. from 3-6 individual mice per group. *P < 0.05; significantly different from the control response by Tukey's multiple comparison tests.

LPA is produced by ATX in the early phase after nerve injury [13,14]; therefore, we administered 2ccPA (10 nmol, i.t. or 100 nmol, i.t.) at 30 min prior to inducing nerve injury. 2ccPA prevented thermal hyperalgesia and mechanical allodynia in a dose-dependent manner at 5 and 7 days after nerve injury (Figure 6). However, 2ccPA (i.t.) injection in naïve C57BL/6 mice had no significant effect on nociceptive latency at 90 min or 1 or 7 days after injection (additional file 1).

### 4.6. Repeated administration of 2ccPA induces analgesia against established neuropathic pain in mice

**Figure 7:**
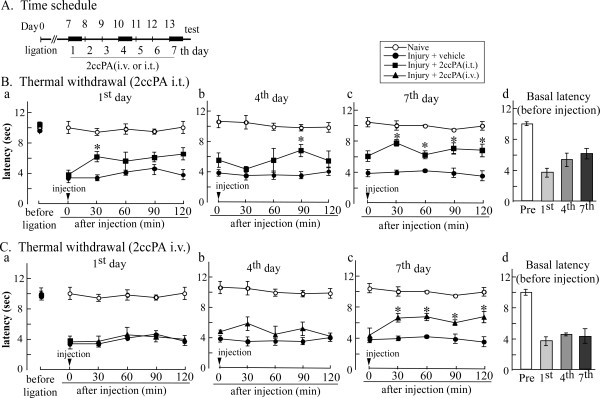
**Repeated administrations of 2ccPA (i.v. or i.t.) induces analgesia against established thermal hyperalgesia in mice with neuropathic pain**. A, Neuropathic pain in mice was induced by partial sciatic nerve injury. Vehicle or 2ccPA (i.v. or i.t.) was injected daily for 7 days, starting 7 days after injury. B, Vehicle or 2ccPA (100 nmol/5 μL 1-3 days and 10 nmol/5 μL 4-7 days, respectively, i.t.) was daily injected for 7 days, starting 7 days after injury. The withdrawal latency was measured at 30, 60, 90, and 120 min after the injection of 2ccPA (i.t.) on the first (a), fourth (b), or seventh day (c). The basal latency was measured before injection of 2ccPA (i.t.) (d). (C) Vehicle or 2ccPA (10 mg/kg, i.v.) was daily injected for 7 days, starting 7 days after injury. The withdrawal latency was measured at 30, 60, 90, and 120 min after the injection of 2ccPA (i.v.) on the first (a), fourth (b), or seventh day (c). The basal latency was measured before injection of 2ccPA (i.t.) (d). All data represent the mean ± S.E. from 3-6 individual mice per group. *P < 0.05 indicates significant difference from the vehicle response by Tukey's multiple comparison test.

We examined the effects of 2ccPA on established neuropathic pain in C57BL/6 mice. In the thermal withdrawal test, mice with sciatic nerve injury exhibited a decreased threshold at day 7. Under these conditions, a single 2ccPA dose (100 nmol, i.t.) significantly increased nociceptive latency at 30 min on the day 7 after injury (considered as day 1), as shown in Figure 7Ba. When 2ccPA (i.t.) was administered daily to the injured mice, the basal latency (before the i.t. injection) time-dependently increased from day 1 to 7, though no significant change was observed on the 7th day (Figures 7Ba-d). A significant increase was observed at 90 min after the 2ccPA injection on the day 4 and at all time points until 120 min on the 7th day (Figure 7Bc). The reason for more pronounced 2ccPA analgesia on the 7th day may be attributed to the fact that there is some, but not significant increase in the basal latency on the 7th day. A single 2ccPA injection (10 mg/kg i.v.) had no effect on thermal hyperalgesia (Figure 7Ca). There was significant analgesia by 2ccPA (i.v.) on the seventh day following daily injections (i.v.), with no change in basal latency throughout the 7 days (Figures 7Ca-d).

**Figure 8:**
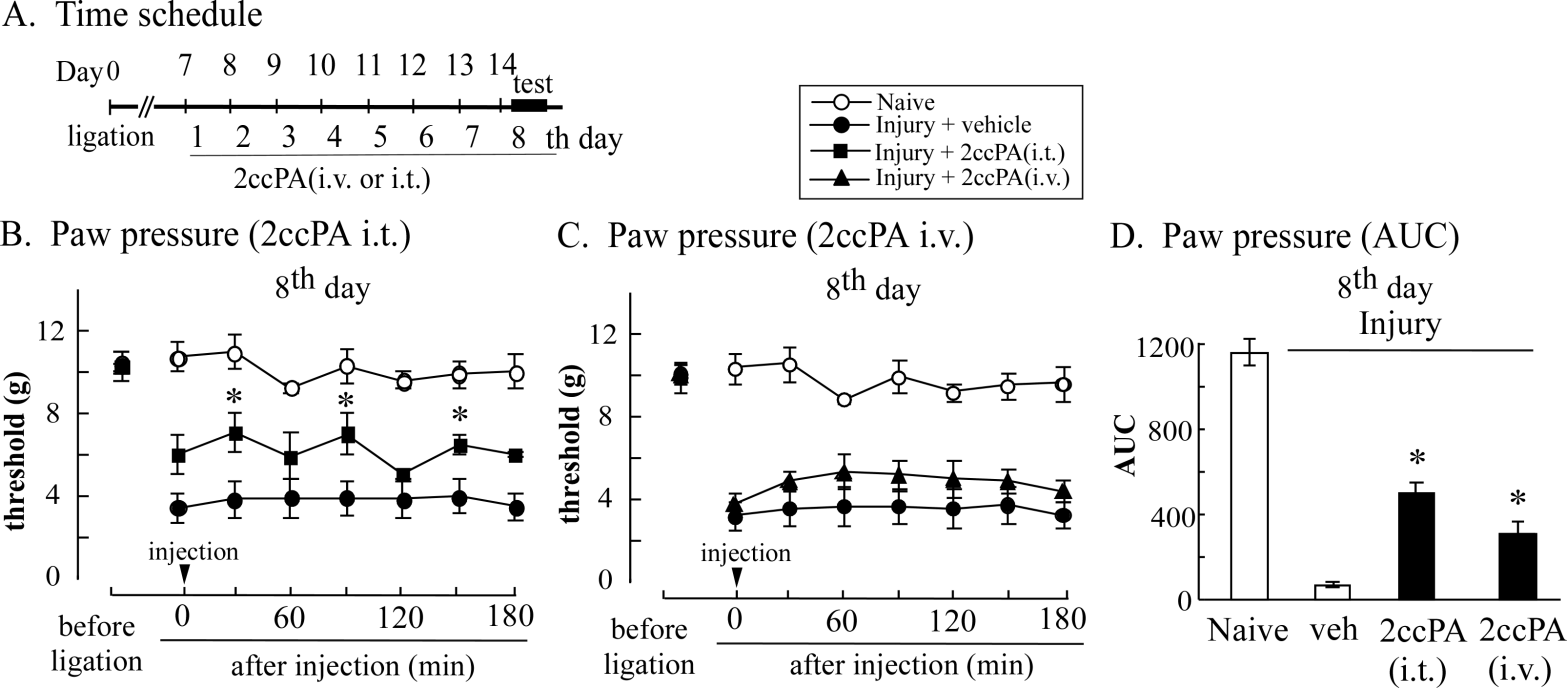
**Repeated administration of 2ccPA (i.v. or i.t.) induces analgesia against established mechanical allodynia in neuropathic pain mice**. Vehicle or 2ccPA (10 mg/kg i.v.) was daily injected for 8 days, starting 7 days after injury (A). The threshold in the paw pressure test was measured at 30, 60, 90, 120, 150, and 180 minutes after 2ccPA application (i.t. or i.v.) on the eighth day (B and C). Quantitative analyses with 2ccPA (i.t. or i.v.) were performed with the area under the curve (AUC) (D). AUC data represent increased thresholds by subtracting the thresholds before injection in vehicle-treated mice. All data represent the mean ± S.E. from 3-6 individual mice per group. *P < 0.05 indicates significant difference from the vehicle response by Tukey's multiple comparison test.

In the paw-pressure test, repeated post-injection (i.t. or i.v.) of 2ccPA yielded similar effects on day 8 (Figure 8). Following repeated i.t. injection, there was an increasing trend in basal latency on day8, while there was no change with i.v. injection. Significant analgesia was also observed at several time points after the 2ccPA i.t. injection but not after the i.v. injection. When the area under the curve (AUC) was evaluated, there was significant analgesia with both i.t. and i.v. injections on day 8 (Figure 8D).

### 4.7. Repeated 2ccPA administration induces analgesia against established neuropathic pain in rats

**Figure 9:**
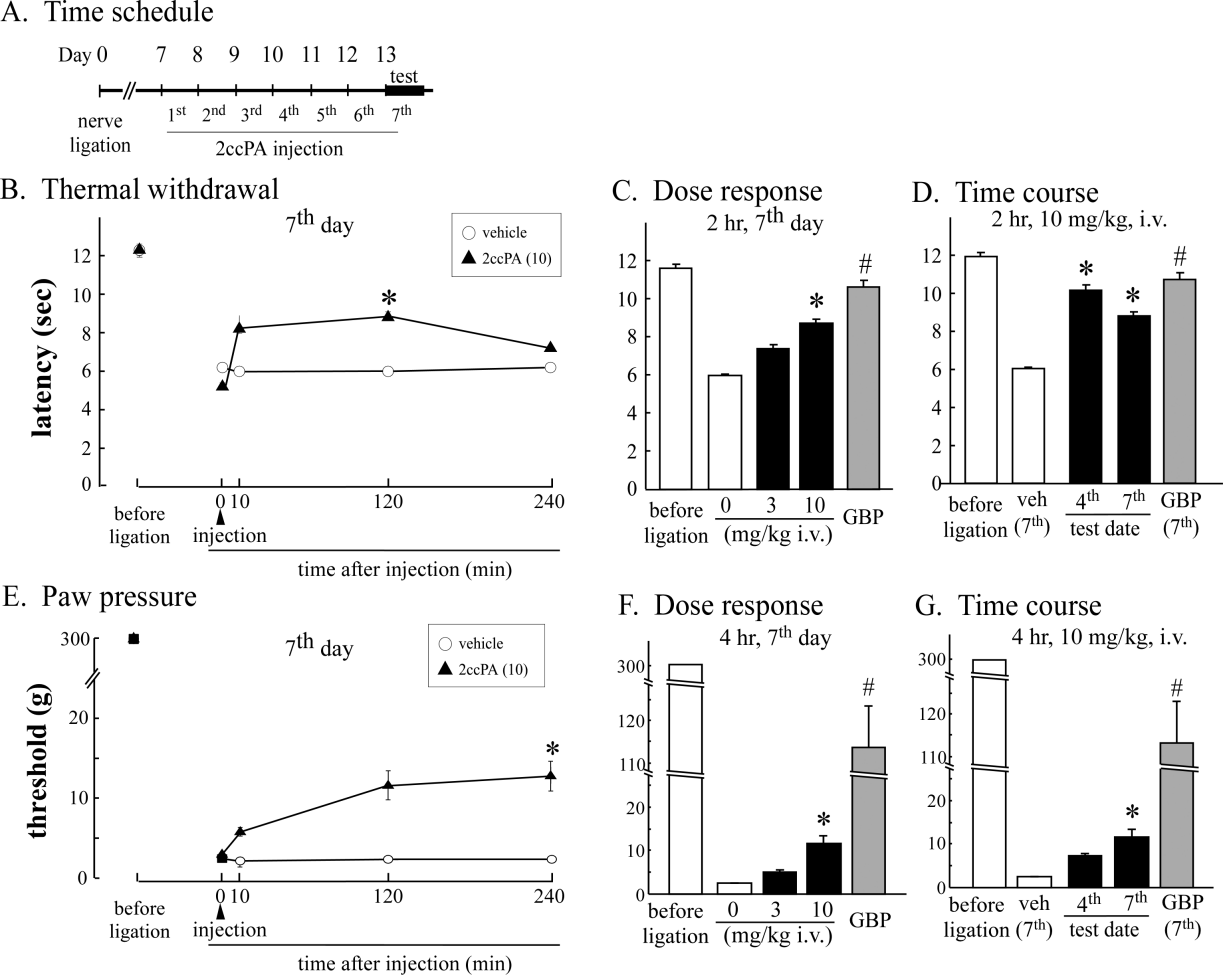
**Effects of i.v. 2ccPA on thermal paw-withdrawal and mechanical allodynia of nerve-ligated rats**. A, The rat sciatic nerve was ligated 7 days prior to i.v. injection of 2ccPA, applied once daily for 7 consecutive days. B, A hind paw was irradiated with infrared light to evoke thermal withdrawal, and the time variation of the withdrawal latency was measured prior to and at 10 min, 2 h, or 4 h after the application of 2ccPA on the seventh day. C, Dose response; 2-h injection of 2ccPA at 3 or 10 mg/kg applied once daily for 7 consecutive days. D, Time course; 2-h injection of 2ccPA (10 mg/kg) applied once daily for 4 or 7 consecutive days. Gabapentin (90 mg/kg i.v.) was applied as a control. *P < 0.05 and #P < 0.05 indicates significant difference from the vehicle response by Dunnett's test and by Student's t-test or Welch's test, respectively. E, The responses of rats to tactile stimulation was tested with 5 von Frey filaments, and threshold filament sizes to evoke paw withdrawal were measured prior to and at 10 min, 2 h, or 4 h after application of 2ccPA on the seventh day. F, Dose response; 4-h injection of 2ccPA at 1, 3 or 10 mg/kg applied once daily for 7 consecutive days. G, Time course; 4-h injection of 2ccPA (10 mg/kg) applied once daily for 4 or 7 consecutive days. Gabapentin (90 mg/kg i.v.) was applied as a control. *P < 0.05 and #P < 0.05 indicates significant difference from the vehicle response by Steel's test and by Wilcoxon test, respectively.

Similar studies to examine the analgesic effects of 2ccPA against established chronic pain were performed using a different chronic-pain model in rats. In the chronic constrictive sciatic nerve injury (CCI) model, the same experimental schedule was performed, as described above (Figure 9). In the thermal withdrawal test, there was significant analgesia at 2 h after i.v. injection of 2ccPA (10 mg/kg) on the seventh day following repeated injections, with no significant change in the basal latency at time 0 min (Figure 9B). There was weak but not significant analgesia at 2 h with 3 mg/kg (i.v.) on the seventh day (Figure 9C), while significant analgesia was observed on the fourth day with 10 mg/kg (Figure 9D). The 2ccPA-induced analgesia was slightly weaker, but comparable to the analgesic effects of gabapentin (90 mg/kg i.v.). Similar results were observed with the paw-pressure test (Figures 9E-9G). In this case, weak but significant analgesia against mechanical allodynia was observed only with 10 mg/kg at 4 h on the seventh day. However, the analgesic effect was much lower than that with gabapentin.

## 5. Discussion

We designed and chemically synthesized the metabolically stabilized derivatives of cPA, to avoid cPA hydrolysis in animals [10]. In 2ccPA, the phosphate oxygen of cPA is replaced with a methylene group at *sn*-2 (Figure 1). This study showed that the effective dose of 2ccPA was almost 10-fold less than that of natural cPA, consistent with other studies [6,7,10]. Differences in chemical stability and/or structural traits might account for the difference in the effective dose of natural cPA and 2ccPA required to achieve analgesia. The specificity of these compounds has been extensively reported [7,10].

Our initial examination demonstrated that intragastral administration of 1 mg/kg of 2ccPA resulted in a remarkable 40% reduction of the somato-cardiac sympathetic C-reflex (additional file 2), suggesting practical stability against gastric digestion as well as rapid gastric absorption of 2ccPA. We examined another carba-cPA, 3ccPA, in which the phosphate oxygen of cPA is replaced with a methylene group at *sn*-3, and found that effective doses of 3ccPA 16:1 were similar to those of 2ccPA for suppression of somato-cardiac sympathetic reflexes (additional file 3). In this report, we demonstrated that cPA and 2ccPA suppressed the supraspinal sympathetic and spinal kinetic reflexes, specifically the C-fiber, but not the A-fiber reflex, in anesthetized animals. This result was consistent with the experiments with mice, in which 2ccPA increased the nociceptive threshold only for Neurometer™ electrical stimulus with 5 Hz, which is supposed to stimulate C-fibers, but not with 250 or 2000 Hz, which are supposed to stimulate Aδ and Aβ-fibers [16]. These results suggest that both cPA and 2ccPA suppress nociceptive responses by primary afferent C-fibers. Both compounds are reported to possess selective and potent ATX inhibitory activities [10]. Endogenous LPA in the peripheral tissues or plasma exerts tonic stimulation of C-fibers, as evidenced by previous findings that LPA injected into the hind paw of mice caused nociceptive flexor responses, partially via substance P release from the nociceptor endings of C-fibers [11,28]. As there was no significant analgesia with i.t.-injected 2ccPA (additional file 1), the inhibitory responses of these compounds are unlikely to be mediated by inhibition of LPA synthesis in naïve mice. However, we cannot exclude the possibility that cPA and/or 2ccPA may have inverse agonist actions on C-fiber nociceptor endings, since they have some weak actions on LPA receptors [7,10].

We also found that 2ccPA exerted anti-nociceptive effects in the formalin test. Formalin-induced characteristic behaviors in phase I are the result of direct C-fiber-evoked excitation, whereas the behaviors in phase II are evoked by repetitive C-fiber stimulation [29,30]. 2ccPA reduced both phase responses, but the inhibition of phase II responses was significant. We speculate that repetitive C-fiber stimulation may cause the ATX-catalyzed production of LPA in the periphery and stimulate C-fibers in an autocrine manner.

It should be noted that 2ccPA attenuated neuropathic pain possibly via the central nervous system. Our initial study revealed that i.t. injection of 2ccPA prevented nerve injury-induced neuropathic pain in mice. This finding is consistent with a series of studies by Ueda and colleagues, in which nerve injury induces LPA production by ATX in the spinal cord and causes neuropathic pain through the LPA_1 _receptor [12,13,31]. The most striking evidence is that repeated administration of 2ccPA through i.t. and i.v. routes produced significant analgesia against established neuropathic pain in mice and rats. The i.t. injection of 2ccPA on day 7 after injury produced weak analgesia against thermal hyperalgesia. More pronounced analgesia was observed when it was given daily by the seventh i.t. injection on day 13 after injury. As there is some, but not significant, recovery of the basal threshold before the seventh injection of 2ccPA, LPA production may occur in the late phase to maintain the neuropathic pain status, as well as at the early phase to trigger the initiating mechanisms [12]. Similar analgesic effects were observed with i.v. injection by the seventh injection on day 13 after injury, though there was no tendency to recover the basal threshold. The difference of basal latency following repeated injections between i.t. and i.v. routes may be related to the fact that there are some residual increases at as late as 120 min in the case with i.t., but not i.v. injections at the 1st and 4th day. Although the lack of elevation in the basal threshold cannot be explained at this time, it seems to occur after repeated i.v. treatments: i.v.-injected 2ccPA-induced analgesia was equivalent to that yielded by the first i.t. injection. We previously reported that cPA and carba derivatives penetrate into the central nervous system through the blood-brain barrier [32]. In the present study, the effective dosage of 2ccPA for mice were about five times higher than that for rats, both for i.v. and i.p. injection, possibly because these two animals exhibit different sensitivities against administered drugs depending on their chemical species [33].

Recently, we demonstrated LPA-induced LPA production; i.t. injection of LPA or the addition of LPA to spinal cord slices markedly increased the LPA level in a time-dependent manner with the peak occurring at 3 h [14,34]. This finding indicates the presence of feed-forward amplification of LPA production in initiating neuropathic pain. As LPA production declines, however, there may be end-product inhibition. Although it is a fascinating mechanism that cPA is a natural ATX inhibitor [10] produced by ATX, it remains to be seen whether the amounts of cPA produced are sufficient to exert this effect.

## 6. Conclusion

Our results indicate that cPA and 2ccPA are potent inhibitors of nociceptive transmission by C-primary-afferents and reverse inflammatory and neuropathic pain. These chemicals may be good candidates for use in clinical pain management.

## List of Abbreviations Used

aCSF: artificial cerebrospinal fluid; ATX: autotoxin; ccPA: carba-cyclic phosphatidic acid; cPA: cyclic phosphatidic acid; i.p.: intraperitoneal; i.v.: intravenous; LPA: lysophosphatidic acid; PBS: phosphate-buffered saline; s.c.: subcutaneously

## Conflicts of interests

The authors declare that they have no competing interests.

## Authors' contributions

YK and JN participated in the experimental designing, collection and analyses of data, and drafted the manuscript in equal contribution. MG performed the statistical analyses and drafted the manuscript. HH participated in the designing of the study, carried out surgical manipulation, data collection, and drafted the manuscript. HM conceived of the study, and participated in its design. TO participated in EPW assay. HU and KM conceived of the study, participated in its design and coordination. All authors read and approved the final manuscript.

## Supplementary Material

Additional file 1 **No effect of 2ccPA (i.t.) injection in naïve mice**. The thresholds were measured at 90 min (A) and on days 1 and 7 (B) after 2ccPA injection, using the thermal withdrawal test. All data represent the mean ± S.E. from 3-6 individual mice per group. Click here for file

Additional file 2 **Time variation of relative C-reflex level after oral administration of 2ccPA (16:1)**. Relative C-reflex levels were measured after oral administration of 2ccPA (16:1) at 1 mg/kg and plotted over time until 120 min. Each data point represents the average four independent measurements, and vertical bar represents S.E. **P < 0.01 and *P < 0.05; significantly different from time 0 by one-way ANOVA, Dunnett's multiple comparison test. Click here for file

Additional file 3 **Effects of i.v. 3ccPA on the somato-carcdiac C-reflex**. Relative C-reflex levels of the somato-cardiac response were measured after i.v. injection of 3ccPA (18:1) at 100 μg/kg (n = 3). Vertical bar represents S.E. **P < 0.01; significantly different from the vehicle by Student's t-test. Click here for file
