# Supplementary figures and images for: Antinociceptive effect of cyclic phosphatidic acid and its derivative on animal models of acute and chronic pain
Source: Mol Pain. 2011 May 14;7:33. doi: 10.1186/1744-8069-7-33 (PMC3113318; doi:10.1186/1744-8069-7-33)

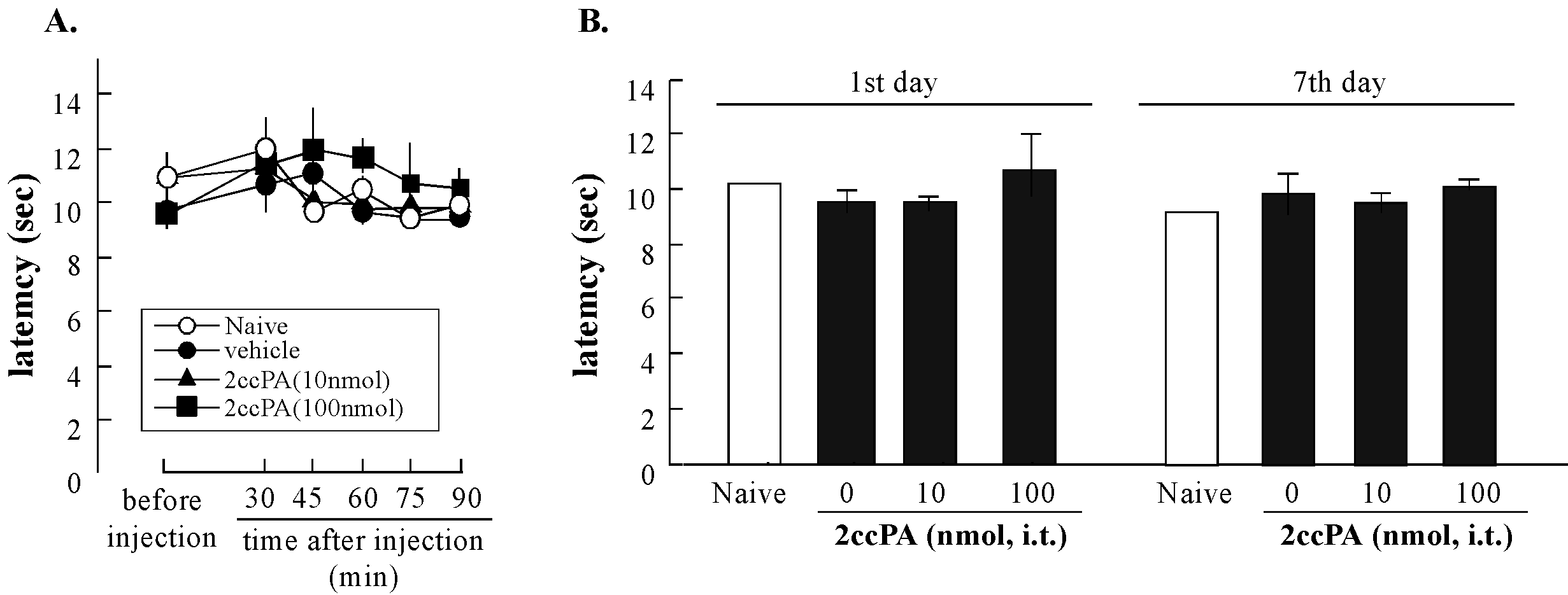

Supplement: Additional file 1 — No effect of 2ccPA (i.t.) injection in naïve mice. The thresholds were measured at 90 min (A) and on days 1 and 7 (B) after 2ccPA injection, using the thermal withdrawal test. All data represent the mean ± S.E. from 3-6 individual mice per group. [file 1744-8069-7-33-S1.TIFF]

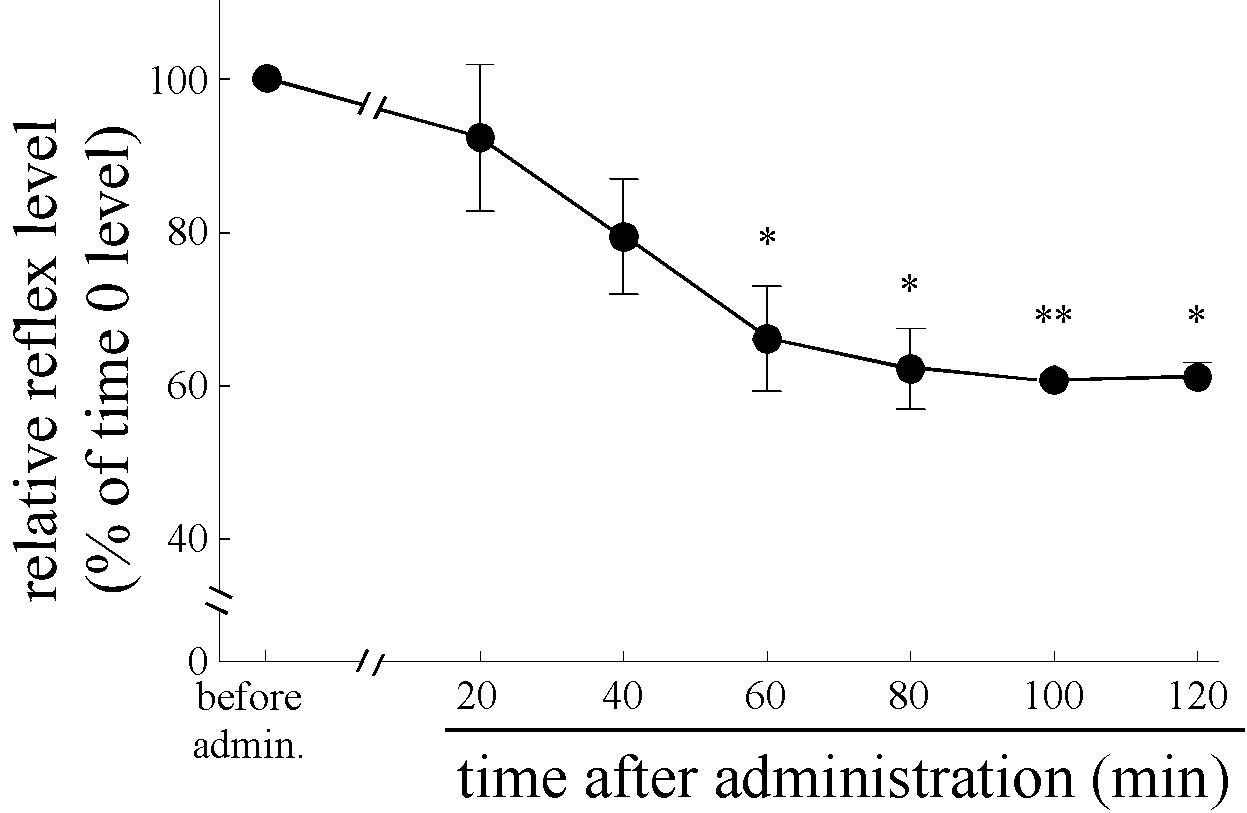

Supplement: Additional file 2 — Time variation of relative C-reflex level after oral administration of 2ccPA (16:1). Relative C-reflex levels were measured after oral administration of 2ccPA (16:1) at 1 mg/kg and plotted over time until 120 min. Each data point represents the average four independent measurements, and vertical bar represents S.E. **P < 0.01 and *P < 0.05; significantly different from time 0 by one-way ANOVA, Dunnett's multiple comparison test. [file 1744-8069-7-33-S2.TIFF]

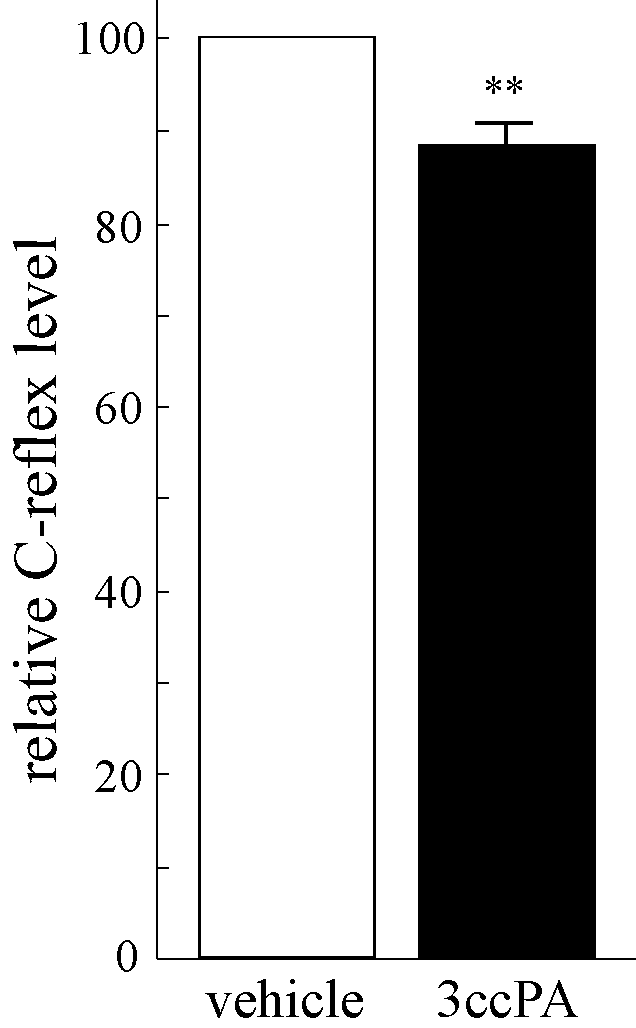

Supplement: Additional file 3 — Effects of i.v. 3ccPA on the somato-carcdiac C-reflex. Relative C-reflex levels of the somato-cardiac response were measured after i.v. injection of 3ccPA (18:1) at 100 μg/kg (n = 3). Vertical bar represents S.E. **P < 0.01; significantly different from the vehicle by Student's t-test. [file 1744-8069-7-33-S3.TIFF]
